# Metabolic and Cardiovascular Comorbidities Among Clinically Stable HIV Patients on Long-Term ARV Therapy in Five Ambulatory Clinics in Lima-Callao, Peru

**DOI:** 10.2174/1874613601812010126

**Published:** 2018-10-17

**Authors:** Jose A Hidalgo, Alberto Florez, Cecilia Agurto, Yvett Pinedo, Rosemarie Ayarza, Lourdes Rodriguez, Alberto La Rosa, Raul Gutierrez

**Affiliations:** 1 Almenara Hospital, Lima, Peru; 2 Grau Hospital, Lima, Peru; 3 Sabogal Hospital, Callao, Peru; 4 Loayza Hospital, Lima, Peru; 5 Vía Libre HIV Clinic, Lima, Lima; 6 MSD Peru Medical Affairs, Lima, Peru

**Keywords:** Metabolic, Cardiovascular comorbidities, HIV patients, ARV therapy, Ambulatory clinics, Lima-Callao

## Abstract

**Background:**

There is scarcity of data about the prevalence of non-AIDS defining comorbidities among stable HIV-infected patients in Peru.

**Objective:**

We aimed to describe the most frequent cardiometabolic comorbidities found among ambulatory adults on ARV in Peru.

**Methods:**

A review of records for patients attending regular visits at 5 clinics in Lima-Callao in January-February 2016 is presented. Patients were adults on ARV for >6 months, with no recent AIDS-defining condition.

**Results:**

Three hundred and five medical charts were reviewed. Most patients were male (73.1%, n=223) with a mean age of 46.0 years. Mean time from HIV diagnosis was 9.41 yrs. and mean duration of ARV was 7.78 yrs. Most patients were on an NNRTI-based first line regimen (76.4%, n=233), and 12.1% (n=37) were on rescue regimens. Median CD4 count was 614.2 cells/µL and the proportion of patients with viral load <40 c/mL was 90.8% (n=277). Most frequent metabolic diagnoses were dyslipidemia (51.5%, n=157), obesity (11.1%, n=34), and diabetes mellitus (7.2%, n=22). Hypertension was diagnosed in 8.9% (n=27). Other diagnoses of cardiovascular disease were documented in 3.3% (n=10). Pharmacologic treatment was prescribed in 91.3% of patients with diabetes or hypertension, but in only 29.3% of patients with dyslipidemia.

**Conclusion:**

A high proportion of metabolic comorbidities was found, with dyslipidemia being the most frequent, followed by obesity and diabetes. In contrast, cardiovascular disease was documented less frequently. Medical treatment was started for only a third of dyslipidemia patients. HIV care policies need to consider proper management of chronic comorbidities to optimize long-term outcomes.

## 1 INTRODUCTION

As access to Antiretroviral therapy (ARV) increases globally and survival improves, cardiometabolic comorbidities are becoming primary contributors of morbidity and mortality among Human Immunodeficiency Virus (HIV) - infected persons [1, 2]. These changes need to be taken into consideration to update policies for HIV care [3].

An increasing frequency of metabolic, cardiovascular and other non-AIDS related comorbidities has been reported initially in Western Europe, North America, and Australia, reflecting improvements in HIV care in those settings [4-14]. More recently, similar findings are being reported from more diverse countries, including those with limited-resources in different continents. Importantly, a significant number of those studies are from Africa and Asia, where AIDS epidemics remain a major cause of morbidity and mortality, and scaling up of ARV is still a work in progress in several countries [15-27].

Several published reports have described this situation in Latin America [28-30] but only one study included data from Peruvian patients. The RAPID study included 417 Peruvian adults who had been on ARV for a mean of only 1.5 years and found a high prevalence of cardiometabolic comorbidities such as dyslipidemia (67.9%) and hypertension (14.9%) [31]. Although the overall findings tend to be similar, some important differences can be identified among different populations, potentially resulting in different health care policy needs.

Given the scarcity of epidemiological data about the prevalence of non-AIDS defining comorbidities among HIV-infected adults in Peru, the aim of this study was to describe the prevalence of metabolic and cardiovascular comorbidities among stable ambulatory adults on ARV.

## 2 MATERIALS AND METHODS

A sample of HIV-infected patients attending five clinics in Lima-Callao for regular follow up visits between January 1 and February 29, 2016 was evaluated. Subjects were adults (>21 years), on ARV for ≥ 6 months, with no current or recent (≤ 6 months) AIDS-defining condition, that had a scheduled appointment for routine outpatient follow up with a physician member of the study team. Medical charts of all consecutive adult patients attending an appointment with a study investigator at the participating clinics during the study period were reviewed. Epidemiological, clinical and laboratory information was collected from medical records of subjects that met eligibility criteria. A target of at least 50 subjects was set for each participating site.

All participating sites are major providers of care for people living with HIV within the two Peruvian public-funded systems (Social Security and Ministry of Health) in Lima and Callao, urban areas where approximately 63% of the HIV-infected Peruvian population reside [32]. Four of the sites provide hospital-based and ambulatory services, and one provides only ambulatory care.

Approval from the institutional review board at each participating site was obtained. No informed consent was obtained, since none of the study procedures involved direct contact with patients and information was handled anonymously.

Definitions of comorbidities such as hypertension [33], diabetes [34], dyslipidemia [35] obesity and excess weight [36] were taken from current well recognized US guidelines. Dyslipidemia was defined as fasting total cholesterol ≥240 mg/dL, and/or fasting triglycerides >150 mg/dL, and/or low high-density lipoprotein (HDL) cholesterol (<40 mg/dL) and/or high low-density lipoprotein (LDL) cholesterol (≥160 mg/dL), or a diagnosis recorded in the medical chart, or indication of lipid-lowering therapy. Arterial hypertension was defined as systolic blood pressure >140mmHg and/or diastolic blood pressure >90mmHg (>135/85mmHg in diabetic patients), or diagnosis recorded in the medical chart or taking antihypertensive drugs. Diabetes mellitus was defined as diagnosis recorded in the chart, or known use of oral anti diabetic drugs or insulin.

Obesity and excess weight were estimated with anthropometric data recorded (height and most recent weight within 6 months of visit). Obesity was defined as Body Mass Index (BMI) >30 kg/m2, and excess weight as BMI between 25 and 30.

In addition, patients were considered to have a history of coronary artery disease if they had one of the following events: known coronary insufficiency, previous myocardial infarction, coronary artery bypass grafting, or coronary angioplasty or stenting. Renal function was estimated with Cockroft-Gault formula (based on most recent serum creatinine within 6 months) to identify cases of renal impairment (creatinine clearance <70 mL/min/1.73m^2^).

Descriptive statistics were applied to summarize epidemiological and clinical findings. Chi-square test with the Fisher´s exact test was used to assess differences among groups.

## 3 RESULTS

Three hundred and five patients were eligible for review (Table **1**). Most of them (73.1%, n=223) were male, and the mean age was 46.0 years (range, 21-79). Sixteen percent of the subjects were 60 years of age and older (n=49). Patients had a diagnosis of HIV infection for a mean of 9.41 years, and were on ARV for a mean of 7.78 years.

Most patients were on a Non-Nucleoside Reverse Transcriptase Inhibitor (NNRTI)-based (nevirapine, efavirenz) first-line regimen (76.4%, n=233), followed by second-line regimens (12.1%, n=37), Protease Inhibitor (PI)-based (lopinavir, atazanavir) first-line regimens (9.2%, n=28), and other combinations for first-line therapy (2.3%, n=7). Integrase Strand Transfer Inhibitor (INSTI) use was very limited, with only 8 patients receiving raltegravir (2.2%). Nucleos(t)ide Reverse Transcriptase Inhibitor (NRTI) use included lamivudine for the great majority (90.5%, n=276), abacavir (50.8%, n=155) –despite HLA testing not being available in the country-, tenofovir (26.2%, n=80) and zidovudine (22.6%, n=69).

Mean CD4 count was 614.2 cells/mL. The large majority (90.8%, n=272) of patients had undetectable viral load (VL <40 c/ml). Seventeen individuals (5.6%) had VL between 40 and 400 and a lower proportion (3.6%, n=11) were in virological failure with a VL >400 c/ml.

The most frequent metabolic diagnoses were dyslipidemia in 51.5% (n=157), obesity (BMI >30) in 11.1% (n=34), and type 2 diabetes mellitus in 7.2% (n=22) of the patients. Excess weight was observed in 41.6% (n=127) of the patients. Among subjects with dyslipidemia who were not receiving lipid-lowering therapy (n=111), a recent (<6 months) complete lipid panel was available for 93 individuals. Most (70/93, 75.3%) of the patients in this group had isolated hypertriglyceridemia, with the majority (40/70, 57.1%) having mild hypertriglyceridemia (150 to 250 mg/dL). The remaining patients had mixed dyslipidemia (total cholesterol elevation and hypertriglyceridemia) in 19 (20.4%), and isolated hypercholesterolemia in 4 (4.3%) cases.

Hypertension was found in 8.9% (n=27) of the patients. Other diagnoses of cardiovascular disease (coronary artery disease, congestive heart failure, cerebrovascular disease) were found in 3.3% (n=10), including a patient that had a diagnosis of acute myocardial infarction. The large majority of patients with diabetes and/or hypertension were receiving treatment for those conditions (91.3%), but only 29.3% (n=46) of those with dyslipidemia were receiving specific pharmacologic treatment.

Renal function impairment (estimated Cr Cl<70) was observed in 19 patients (6.2%) and included two cases of subjects with end-stage renal disease not related to HIV. Seventeen remaining patients had milder degrees of deterioration, with 14 of them having a risk factor, such as older age, hypertension or diabetes mellitus. Only four of these 17 patients had a history of current exposure to tenofovir.

Distribution of obesity and excess weight was very similar within subgroups stratified by gender, age (Table **2**), duration, or type of ARV (Table **3**). Dyslipidemia, diabetes mellitus, hypertension, appeared related to older age (*p* values between 0.006 and <0.001). Dyslipidemia showed also an association with longer duration of ARV (*p* = 0.005). The strength of association with longer duration of ARV was weaker for diabetes mellitus (*p* = 0.071) and hypertension (*p* = 0.061). Renal function impairment was associated with older age (>50 years) (*p* < 0.001) (Tables **2** and **3**).

In summary, the analysis of cardiometabolic comorbidities versus age, gender, duration and type of antiretroviral therapy, revealed that: 1) excess weight and obesity were highly prevalent but did not differ among groups for the variables analyzed; 2) the prevalence of dyslipidemia, hypertension, renal function impairment and diabetes mellitus was higher among those 50 years or older when compared to those under 50 years of age; and there was a trend toward those with longer (>5 years) duration of ARV having dyslipidemia, hypertension or diabetes mellitus; 3) cardiovascular disease was observed in a low number of individuals precluding analysis of associations among groups.

## 4 DISCUSSION

In this sample of HIV-infected adults that were clinically stable, had been receiving predominantly NNRTI-based initial antiretroviral therapy for several years, and had a high proportion of virologic and immunologic control, a high proportion of metabolic comorbidities were found, with dyslipidemia, obesity and type-2 diabetes being the most frequent. To our knowledge, this is the first study that assesses cardiometabolic comorbidities among an adult population that has received ARV for a median longer than three years in Peru. The mean time on ARV was 7.78 years in our study versus 1.5 years in RAPID, and 3 years in a recent study from a different Lima Hospital [31, 37]

The predominance of male participants reflects the local epidemiology of HIV infection. Our sample had a higher proportion of persons older than 60 years in comparison to previous local reports [32, 38].

While the global trend is to observe a growing proportion of patients having metabolic and cardiovascular comorbidities, differences have been reported in various settings. For example, frequencies of hypertension appear to be much higher in countries like Puerto Rico, Malawi, Uganda and Switzerland (Table **4**). A comparison of the main findings from this and previous studies, including some from Latin American countries and some from other parts of the world is presented in Table **4**. As a reference, we included studies from populations not infected with HIV. Differences in study designs limit the comparisons. However, some similarities can be observed in Latin American studies in terms of demographic composition (gender, age) and frequency of metabolic comorbidities [30, 31, 39]

In this study, obesity and excess weight were highly frequent and with similar distribution within subgroups divided by age, gender, type and duration of ARV, perhaps reflecting the importance of non –HIV related factors such as diet and lifestyle as seen in the general population.

Dyslipidemia, diabetes, and hypertension showed an association between older age and to a lesser extent to longer duration of ARV. This observation suggests a possible association of those metabolic comorbidities with the long-term use of regimens combining NRTI plus either NNRTI or PI. Protease inhibitors are well known to be associated with dyslipidemia and alterations in glucose metabolism, and efavirenz is associated with dyslipidemia [40-42]. While patients with HIV and dyslipidemia may benefit from lipid-lowering therapy according to current ASCVD guidelines [43], the field is evolving towards the preferential use of more lipid friendly antiretroviral drugs such as integrase inhibitors, including switch strategies [44].

There is abundant data on the importance of cardiovascular complications among persons living with HIV in multiple settings [45, 46], but scarce data is available from Peru [44]. In our study, the frequency of cardiac comorbidities documented in the medical charts was low. A Peruvian series of HIV cases showed a low number of cardiovascular hospitalizations with 26 patients having such events over 16 years of care at the largest hospital in the country [47]. A recent evaluation of two cardiovascular risk assessment scores in a Peruvian population of HIV-positive individuals found low percentages of patients with high cardiovascular risk according to the Framingham score and the PROCAM score (3.6 and 5.4%, respectively) [48]. In the RAPID study [31], the Framingham risk score was 6.1% for Peruvian participants versus 10.4% for the entire Latin American group. A similar estimate in a German HIV-positive population was 7.9% using the Framingham score [7]. It appears that in Peruvian HIV-infected adults, cardiovascular complications are less frequent than in other Latin American and industrialized countries, although metabolic comorbidities are of similar frequency (Table **4**). This could also be the product of medical inertia preventing timely screening for cardiovascular disease before the onset of symptoms.

An important limitation of this study is that no laboratory or additional exams were requested due to its observational nature. Information on transmission risk group was unavailable due to the nature of the study. Since information was collected from medical records and abdominal circumference was not routinely measured, it was not possible to estimate the frequency of metabolic syndrome. In addition, a number of patients did not have complete laboratory values or complete diagnostic workup, potentially resulting in an underestimation of the prevalence of cardio metabolic comorbidities. While we identified a low proportion of patients on medical therapy for dyslipidemia, we did not determine whether all subjects met current criteria for pharmacologic treatment of dyslipidemia.

The objective of the study was to evaluate the frequency of cardiovascular and metabolic comorbidities. Consequently, other complications such as neoplasia, chronic infections, substance abuse and mental illness were beyond its scope.

The frequency of chronic kidney disease was also within the vicinity of a recently reported global estimate of 6.4% for HIV-infected persons (versus 6.2% as reported in this study) [49].

The prevalence of cardiometabolic diseases evaluated in this study seems to be similar to those reported for Peruvian general population as shown in Table **5** [50-53]. There is a need for a prospective study that would include comprehensive active diagnostic evaluations in order to better ascertain the prevalence of cardiometabolic comorbidities and cardiovascular risk among adults living with HIV in Peru who are on ARV.

There is growing agreement on the need to optimize long-term HIV care by implementing strategies for timely diagnosis and management of these comorbidities [54-57]. A strategic approach for diagnosing and managing common comorbidities is needed [58, 59] tailored to the resources available at each setting.

Health care systems, in particular those in resource-limited settings, would need to address the most efficient approach to provide proper care for cardiometabolic comorbidities, with HIV care physicians acquiring the necessary knowledge and experience versus setting up an efficient referral system [60].

## CONCLUSION

**Fig. (1):**
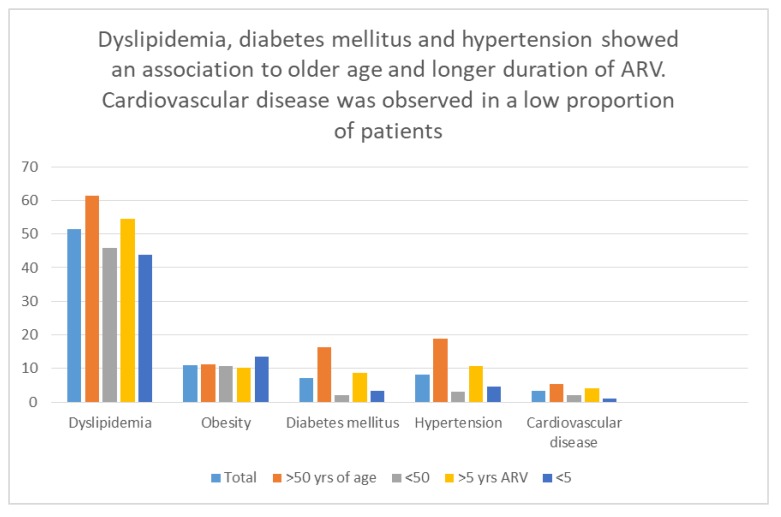
Graphical abstract.

In this sample of middle-aged, clinically stable HIV infected adults, receiving predominantly NNRTI-based initial antiretroviral therapy, a high proportion of metabolic comorbidities were found, with dyslipidemia, obesity and type 2 diabetes mellitus being the most frequent, showing an association to longer duration of ARV and older age (Fig. **1**). Most patients with dyslipidemia were not receiving lipid-lowering therapy. A relatively low frequency of cardiovascular disease was found in comparison to studies from other regions, including Latin America. These results highlight the importance of screening for comorbidities and providing timely and appropriate interventions to address patient’s comorbidities in order to improve long-term outcomes of HIV care in resource-limited settings.

## Figures and Tables

**Table 1:** Demographic and clinical characteristics of studied population.

**Mean Age** (years+/-sd)	46.0+/-11.9
<50 years (n, %)	194, 63.6
≥50 years (n, %)	111, 36.4
**Male (**n, %)	223, 73.1
**Mean time since HIV diagnosis** (years+/-sd)	9.41 +/- 5.11
**Mean time on ARV therapy** (years+/-sd)	7.78 +/- 4.28
**ARV therapy regimen** (n, %)	
1^st^ line NNRTI-based	233, 76.4
1^st^ line, PI-based	28, 9.2
rescue regimen	37, 12.1
other regimens	7, 2.3
**Viral load** (n, %)	
<40 copies/µL	277, 90.8
40-400 copies/µL	17, 5.6
>400 copies/µL	11, 3.6
**Mean CD4 cell count** (cells/µL +/- sd)	614.2 +/- 295.8

**Table 2:** Frequency of metabolic and cardiovascular comorbidities observed by age group and sex.

**Comorbidities**	**Total**	**Men**	**Women**	***p* value**	**< 50 years**	**≥ 50 years**	***p* value**
		**(n=223)**	**(n=82)**		**(n=194)**	**(n=111)**	
**Dyslipidemia, n (%)**	157 (51.5)	111 (49.8)	46 (56.1)	0.197	89 (45.9)	68 (61.3)	0.006
**Obesity, n (%)**	34 (11.1)	25 (11.2)	9 (11.0)	0.568	22 (11.3)	12 (10.8)	0.524
**Excess weight, n (%)**	127 (41.6)	99 (44.4)	28 (34.1)	0.068	76 (39.2)	51 (45.9)	0.150
**Diabetes mellitus, n (%)**	22 (7.2)	15 (6.7)	7 (8.5)	0.373	4 (2.0)	18 (16.2)	< 0.001
**Hypertension, n (%)**	27 (8.9)	21 (9.4)	6 (7.3)	0.375	6 (3.1)	21 (18.9)	< 0.001
**Cardiovascular diseases, n (%)**	10 (3.3)	6 (2.7)	4 (4.9)	0.267	4 (2.1)	6 (5.4)	0.108
**Impairment of renal function, n (%)**	19 (6.2)	10 (4.5)	9 (10.9)	0.039	3 (1.5)	16 (14.4)	<0.001

**Table 3:** Frequency of metabolic and cardiovascular comorbidities observed by type of ARV and duration of treatment.

**Comorbidities**	**Total**	**NNRTI-based**	**PI-based/ rescue**	***p* value**	**< 5 years**	**≥ 5 years**	***p* value**
		**(n=233)**	**(n=72)**		**(n=89)**	**(n=216)**	
**Dyslipidemia, n (%)**	157 (51.5)	122 (52.4)	35 (48.6)	0.336	39 (43.8)	118 (54.6)	0.005
**Obesity, n (%)**	34 (11.1)	24 (10.3)	10 (13.9)	0.258	12 (13.5)	22 (10.2)	0.259
**Excess weight, n (%)**	127 (41.6)	102 (43.8)	25 (34.7)	0.109	36 (40.5)	91 (42.1)	0.444
**Diabetes mellitus, n (%)**	22 (7.2)	19 (8.2)	3 (4.2)	0.191	3 (3.4)	19 (8.8)	0.071
**Hypertension, n (%)**	27 (8.9)	23 (9.9)	4 (5.6)	0.188	4 (4.5)	23 (10.6)	0.061
**Cardiovascular diseases, n (%)**	10 (3.3)	6 (2.6)	4 (5.6)	0.189	1 (1.1)	9 (4.2)	0.158
**Impairment of renal function, n (%)**	19 (6.2)	12 (5.1)	7 (9.7)	0.131	4 (4.5)	15 (6.9)	0.301

**Table 4:** Comparison of study results *versus* other published studies.^*^

	**Present Study**	**Wilson** **2012 [** **30** **]**	**Garcia 2016 [** **8** **]**	**Rodriguez Diaz 2016 [** **8** **]**	**RAPID/** **LATAM 2010 [** **31** **]**	**RAPID/** **Peru 2010 [** **31** **]**	**Muyanja 2016 [** **22** **]**	**Divala 2016 [** **17** **]**	**Esser 2013 [** **7** **]**	**Hasse 2010** [11]	**CARMELA** **2008 [** **61** **]**	**HCH/SOL** 2012 [62]
**HIV status**	Positive	Positive	Positive	Positive	Positive	Positive	Positive	Positive	Positive	Positive	Negative	Negative (men)
**Setting**	Lima – Callao, Peru	Santiago, Chile	Basque Country, Spain	Puerto Rico	South America	Lima, Peru	Mbarara, Uganda	Zomba, Malawi	Leipzig, Germany	Switzerland	Major cities in LATAM	Hispanics USA
**Sample size (n)**	305	121	224	250	4010	417	250	952	803	8444	11550	15079
**Mean Age, years**	46.0	46	48.8	47.9	41.9	39.1	36	43	44.2	45	NR	NR
**Male (%)**	73.1	76.8	74.6	71.6	73.9	70.7	32	28.3	83.4	70.8	NR	NR
**Median time on HAART, years**	7.78	10	NR	3.4	2.91	1.5	2.6	4	NR	6.4	N/A	N/A
**% VL < 50**	90.8	85.2	93.3	NR	NR	NR	NR	NR	78.2	81	NR	NR
**Mean CD4 count**	614.2	602	605	723.8	417	255	466	NR	461	528	N/A	N/A
**Dyslipidemia (%)**	51.5	39.6	37.9	60.8	80.2	67.9	NR	28.7 ↑Tg, 15.5 ↑Chol	64.3	NR	14	52
**Obesity (%)**	11.1	NR	8.5	19.2	7.9	6.5	12	3.8	NR	NR	23	37
**Diabetes mellitus (%)**	7.2	4.1	21.9	19.6	3.3	1.7	NR	4.1	5.1	4.1	7	17
**Hypertension (%)**	8.85	4.95	21.9	39.6	31.5	14.6	5.2	23.7	21	56.3	18	25
**Other (%)**	Renal impairment 6.2	---	Hep C 51.3	EtOH abuse 48.8	---	---	---	---	CVD 10.1	Hep C 22.7	---	---
**Metabolic syndrome (%)**	NR	NR	NR	NR	20.2	13.7	23.2	NR	NR	NR	20	NR

NR=Not Reported; N/A=Not Available; ↑Tg, hypertriglyceridemia; ↑Chol, hypercholesterolemia^*^: Selected studies in HIV-positive populations were considered for: 1) published during the current decade, 2) represented diverse settings from Latin America, Europe and Africa, and 3) evaluated similar populations and variables.

**Table 5:** Comparison of study results to estimates for the Peruvian general population.

**Comorbidity**	**Present study**	**INEI 2015 [** **53** **]**	INS 2006 [50]	Alvarez-Dongo 2012* [51]	**Seclen 2015**[52]
Dyslipidemia (%)	51.5	-	47.6	-	-
Obesity (%)	11.1	17.8	-	19.8	-
Excess weight (%)	41.6	35.5	-	42.5	-
Diabetes mellitus (%)	7.2	2.9	-	-	7.0
Hypertension (%)	8.9	12.3	-	-	-

* Data shown for subjects with ages between 30 and 59 years for comparison with our series.
